# Sinonasal Squamous Cell Carcinoma Mimicking a Brain Abscess: Report of a Unique Case

**DOI:** 10.7759/cureus.68687

**Published:** 2024-09-05

**Authors:** Abhishek Patil, Prem Durai, Preeti Shetti, Teena Desai, Suprita Teli

**Affiliations:** 1 Neurosurgery, Jawaharlal Nehru Medical College, Belgaum, IND; 2 Otolaryngology - Head and Neck Surgery, Jawaharlal Nehru Medical College, Belgaum, IND; 3 Anesthesiology, Jawaharlal Nehru Medical College, Belgaum, IND

**Keywords:** frontal lobe abscess, paranasal sinuses, sinonasal cancer, squamous cell carcinoma, staphylococcus aureus

## Abstract

Sinonasal cancers are rare tumors with squamous cell carcinoma (SCC) being one of the more common histological subtypes. These carcinomas typically invade the sinus cavity from which they originate, progressively eroding surrounding bony structures and extending to adjacent anatomical regions. In rare instances, they may breach the posterior or superior walls to invade the anterior cranial fossa (ACF) and frontal lobes. The normal flora of the nasal cavity and paranasal sinuses includes *Staphylococcus aureus*, *Staphylococcus epidermidis*, α- and γ-streptococci, *Propionibacterium acnes*, and aerobic diphtheroids. To our knowledge, cases of sinonasal malignancies extending into the frontal sinus and ACF, leading to a frontal lobe abscess caused by these organisms, have not been well-documented. We present a case of a 36-year-old male who underwent surgery for a right frontal brain abscess caused by *Staphylococcus aureus*. Histopathological analysis of the abscess wall revealed moderately differentiated SCC arising from the paranasal sinuses, highlighting a rare and intriguing presentation of this disease.

## Introduction

Sinonasal malignancies encompass a diverse group of tumors, some of which are specific to the nasal region. These cancers are rare, accounting for less than 1% of all malignancies, and have a higher prevalence in females compared to males, with a reported ratio ranging from 1:4 to 1:3. Early in their course, these tumors often present with subtle or non-specific symptoms and are frequently misdiagnosed as benign conditions such as rhinosinusitis. This leads to a delay in diagnosis, with most cases identified only at more advanced stages when symptoms from local invasion become apparent. The most commonly affected sites include the maxillary sinus, nasal cavity, ethmoid sinus, frontal sinus, and sphenoid sinus. In some cases, lymph nodes in the inguinal, retroperitoneal, and mediastinal regions may also be involved [1-3].

Sinonasal cancers represent approximately 3% of all head and neck malignancies, with an estimated incidence of one case per 100,000 people worldwide each year [1]. The average age of diagnosis typically falls between 50 and 60 years. Squamous cell carcinoma (SCC) is the most prevalent histological subtype, comprising 50% to 80% of sinonasal malignancies, while intestinal-type adenocarcinomas account for 10% to 20% of cases [4]. These percentages can vary depending on geographical location, with European countries reporting the highest incidences of both types of sinonasal tumors.

The normal bacterial flora of the sinonasal cavity includes *Staphylococcus aureus*, *Staphylococcus epidermidis*, *Escherichia coli*, *Citrobacter*, *Proteus*, *Streptococcus pneumoniae*, *Haemophilus influenzae*, and anaerobic species such as *Bacteroides*. Typically, a bacterial abscess in the sinonasal region is caused by one of these organisms [5,6]. However, the abscess observed in this particular case exhibits a unique and noteworthy characteristic.

## Case presentation

A 36-year-old male patient working as a bank employee presented with complaints of a holocranial, throbbing headache that had been worsening over the past one month. He also reported having moderate to high-degree fever that had been present for the past three days. The patient experienced one episode of seizure on the day of his admission. There was no history of trauma, or bladder or bowel disturbances and the patient did not have any other significant co-morbidities, except that he was a smoker for the past 10 years. Upon examination, the patient had no neurological deficits.

**Figure 1:**
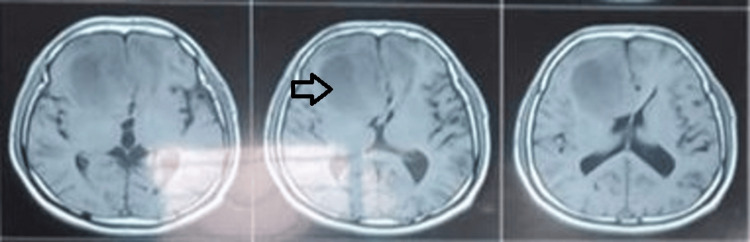
Depicting pre-operative MRI brain (T1 images); isointense intra-axial space-occupying lesion in the right frontal lobe.

**Figure 2:**
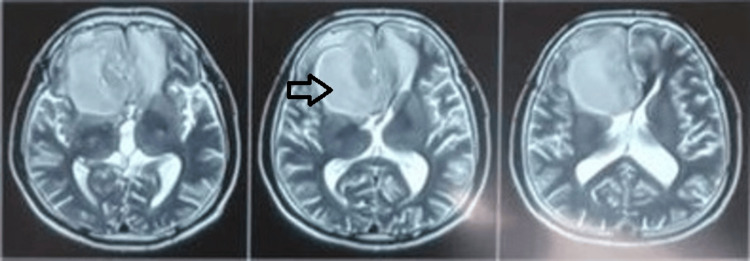
Depicting pre-operative MRI brain (T2 images); heterointense intra-axial space-occupying lesion in the right frontal lobe.

Routine pre-operative blood investigations were within normal limits, and a chest X-ray did not reveal any significant abnormalities. Magnetic resonance imaging (MRI) of the brain identified an intra-axial space-occupying lesion in the right frontal lobe, exerting a mass effect on the corpus callosum with subfalcine herniation to the left side. The lesion appeared isointense on T1-weighted images (Figure 1), heterointense on T2-weighted images (Figure 2) and showed uniform diffusion restriction.

**Figure 3:**
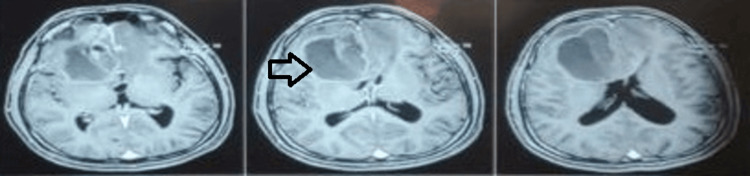
Depicting pre-operative contrast-enhanced T1 MRI images; ring enhancement pattern with extension into the right frontal and ethmoid sinuses.

Contrast-enhanced T1 images revealed a ring enhancement pattern, with the abscess extending into the right frontal and ethmoid sinuses (Figure 3). A differential diagnosis of a bacterial abscess versus a fungal granuloma, such as mucormycosis, was considered.

**Figure 4:**
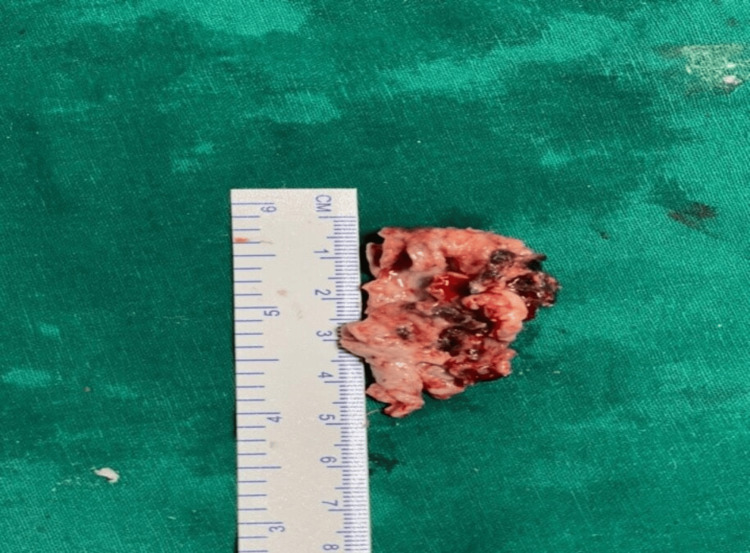
Depicting the excised abscess wall.

The patient underwent bifrontal craniotomy with complete excision of the frontal abscess, along with extradural anterior cranial fossa base repair and frontal sinus exteriorization. Intraoperatively, the abscess was found to have a thick, grayish-red wall and contained purulent, non-foul-smelling yellowish pus. An attempt was made to completely excise the abscess wall (Figure 4).

**Figure 5:**
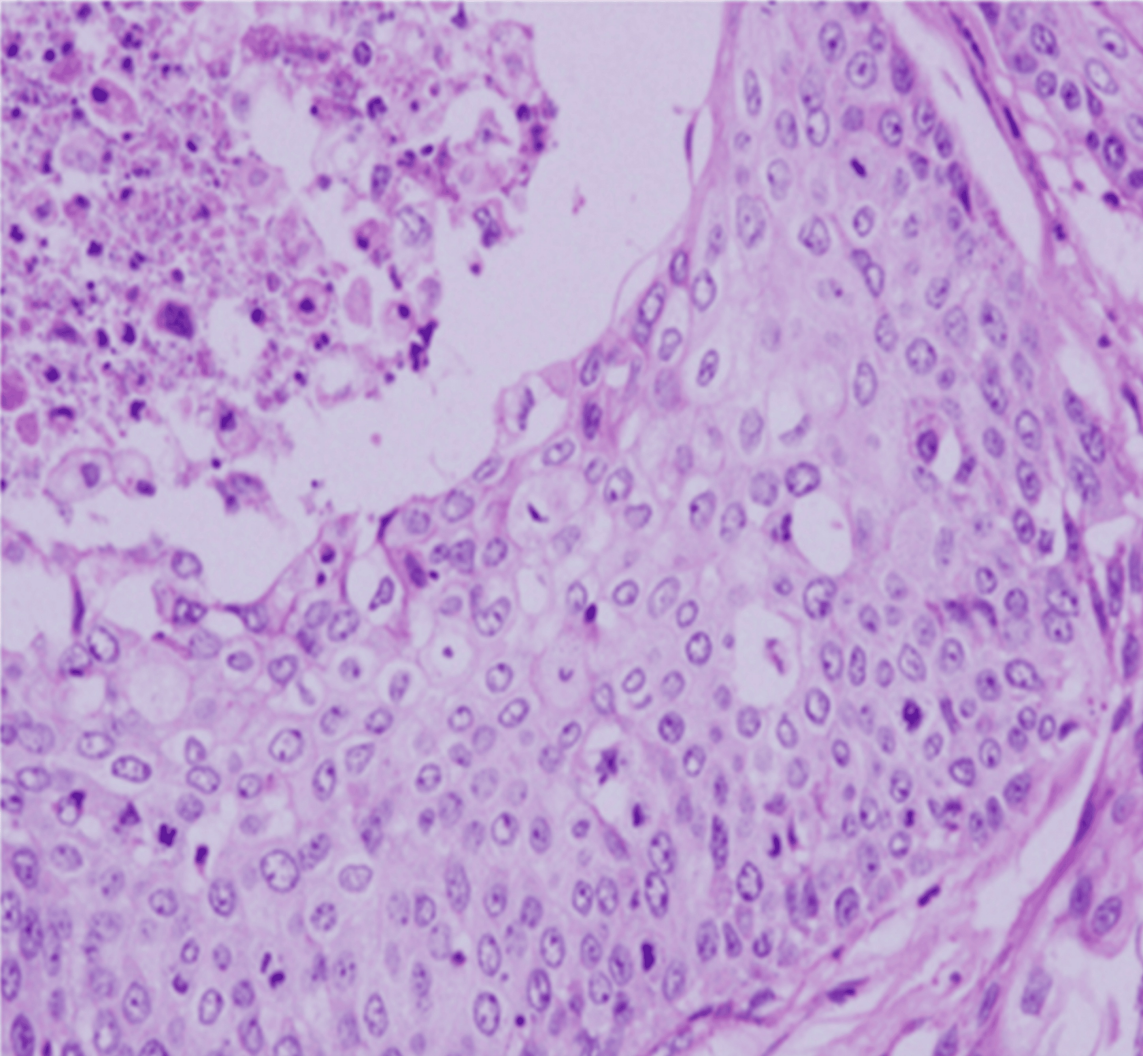
Depicting the histopathological features of the excised wall; highly cellular and invasive epithelial neoplasm organized in sheets (hematoxylin and eosin stain).

**Figure 6:**
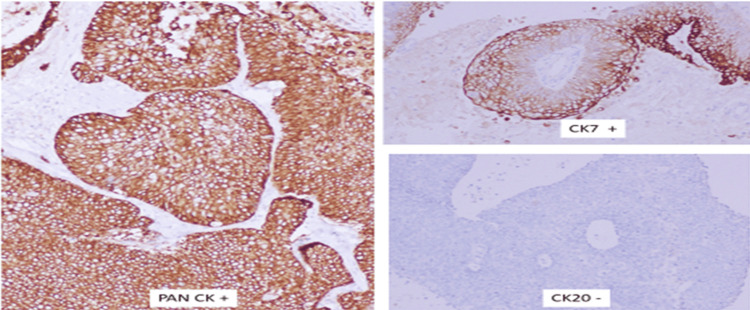
Depicting immunohistochemical analysis; positive staining for pancytokeratin, focal positivity for cytokeratin-7, and negative staining for cytokeratin-20.

Histopathological examination of the excised wall revealed a highly cellular epithelial growth organized in papillae, lobules, sheets, and nests. The tumor displayed invasive characteristics, with polygonal cells exhibiting pleomorphic vesicular nuclei and moderate cytoplasm. Focal areas of keratin pearl formation and active mitosis were observed. The stroma showed significant necrosis and hyalinization, with a notable lymphocytic infiltrate in the interstitial spaces (Figure 5). Immunohistochemical analysis demonstrated positive staining for pancytokeratin, focal positivity for CK, and negative staining for CK20 (Figure 6).

**Figure 7:**
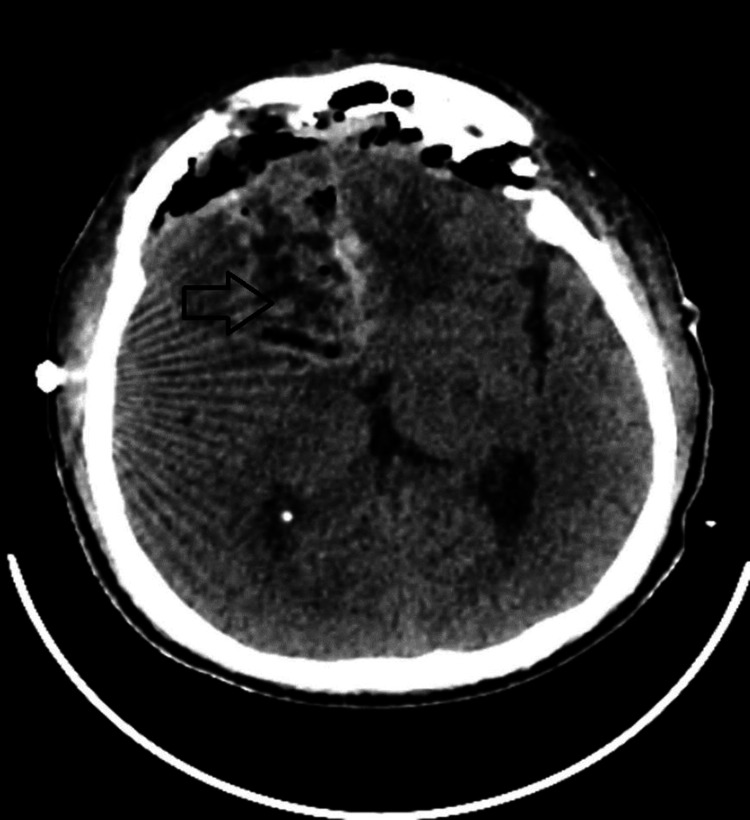
Depicting post-operative CT brain (plain); complete excision of the abscess wall and no operative site hematoma.

Postoperatively, the patient exhibited no neurological deficits. A follow-up CT scan showed mild swelling in the corpus callosum and bifrontal lobes, with no evidence of hematoma at the operative site (Figure 7). Culture and sensitivity testing of the pus drained from the abscess confirmed the presence of *Staphylococcus aureus*, which was sensitive to vancomycin. The patient showed significant symptomatic improvement and was discharged on antibiotic therapy. Unfortunately, the patient lost follow-up after receiving the histopathological report from the hospital and never came back in spite of being advised to take adjuvant radio-chemotherapy.

## Discussion

SCC is the most prevalent type of cancer arising in the sinuses and nasal cavity, representing approximately 50% of all causal types of sinonasal malignancies including adenocarcinoma (12.6%), melanoma (6.6%), esthesioneuroblastoma (6.3%), adenoid cystic carcinoma (6.2%), and sinonasal undifferentiated carcinoma (3.1%). SCC is often detected at an advanced stage, as patients typically present with symptoms related to invasion of adjacent structures (54.5%) or, less commonly, with signs of distant metastasis (14.9%). Early-stage diagnosis is relatively rare, occurring in about 30.6% of cases. The maxillary sinus is the most frequent site of origin for sinonasal SCC, followed by the nasal cavity and ethmoidal complex [1-3].

Sinonasal SCCs are particularly notorious for their aggressive behavior, characterized by the rapid destruction of surrounding bony structures and invasion into neighboring anatomical areas, including adjacent sinuses, the orbital wall, the infratemporal fossa, and the skull base [4]. As a result, patients often experience a range of symptoms depending on the structures affected by the tumor. Common clinical manifestations include nasal obstruction, epistaxis (nosebleeds), facial pain, and a persistent nasal discharge. In cases of advanced disease, symptoms may become more specific, reflecting the extent of local invasion, such as visual disturbances, proptosis, or cranial nerve deficits.

The prognosis for patients with sinonasal malignancies is generally poor, and survival rates vary significantly depending on the histological type and stage at diagnosis. SCC is associated with an intermediate survival rate, with an estimated five-year survival of around 53%. The aggressive nature of SCC, combined with its tendency to be diagnosed at a late stage, contributes to the overall poor prognosis [7].

The nasal cavity's normal flora includes bacteria such as *Staphylococcus aureus*, *Staphylococcus epidermidis*, and *Streptococcus pneumoniae*. While sinonasal malignancies with intracranial extension have been reported, particularly in cases of sinonasal undifferentiated carcinoma, brain abscesses related to cancer are most commonly associated with complications following radiation therapy. There have also been rare cases of brain abscesses linked to ethmoidal and frontal sinus osteomas [1-3]. However, to date, there have been no documented instances of sinonasal SCC mimicking a brain abscess caused by Staphylococcus aureus in the medical literature.

In this unique case, the presentation of a brain abscess led to the discovery of an underlying sinonasal SCC, a scenario not previously reported. The presence of *Staphylococcus aureus* in the abscess is particularly noteworthy, as it highlights a rare but critical diagnostic challenge. The differentiation between a primary brain abscess and one secondary to an underlying malignancy is crucial, as it directly impacts the treatment approach and prognosis.

Unfortunately, the patient’s failure to attend follow-up appointments hindered the continuation of care, including the administration of necessary adjuvant chemoradiation therapy. This lapse in treatment underscores the importance of close follow-up and patient compliance in managing such complex cases. As the next step, we proposed performing a biopsy via the transnasal route to reconfirm the diagnosis. This procedure would have provided additional histopathological confirmation, ensuring that the initial findings were accurate and guiding further treatment decisions.

## Conclusions

Sinonasal squamous cell carcinoma (SCC) extending into the frontal sinus and anterior cranial fossa, resulting in a frontal lobe abscess caused by organisms typically found in the paranasal sinuses represents an exceptionally rare occurrence. To the best of our knowledge, there have been no documented cases of sinonasal SCC mimicking a brain abscess caused by *Staphylococcus aureus* in the global medical literature. Despite its rarity, this case provides valuable insights that can refine differential diagnoses and potentially improve patient outcomes in similar clinical scenarios.
